# ADULT PROTECTIVE SERVICES PROFESSIONALS WITH CLIENTS AND FAMILIES AT THE END OF LIFE: CHALLENGES AND SUPPORT NEEDS

**DOI:** 10.1093/geroni/igae098.2920

**Published:** 2024-12-31

**Authors:** Wei- Lin Xue, Joy Ernst

**Affiliations:** Purdue University, West Lafayette, Indiana, United States; Wayne State University, Detroit, Michigan, United States

## Abstract

This study examines the experiences of Adult Protective Services (APS) workers confronting elder mistreatment at the end of life, a period of heightened vulnerability and increased abuse risk. While previous research highlights the necessity of multifaceted strategies to tackle elder abuse, the specific challenges faced by APS professionals in these critical circumstances remain underexplored. The research team conducted seven focus groups and one individual interview with APS workers and those closely associated with them. The 36 participants, including social workers, nurses, and other allied professionals, provided information on the types of elder abuse encountered, challenges or barriers in serving these clients, and the personal or professional effects these cases have on them. The qualitative analysis revealed that caregiver neglect and financial exploitation are the most frequent forms of abuse, along with self-neglect, emotional, physical, and sexual abuse. APS professionals face significant challenges such as balancing client self-determination, navigating family issues, accessing adequate care levels, addressing service refusal, and coping with a lack of services. The personal and professional impacts on APS workers include heightened empathy, trauma from client deaths, and a noted lack of preparation for such events. The workers also identified the need for increased interagency collaboration, organizational support, mental, and emotional support, and peer support to assist APS workers in dealing with clients at the end of life. These insights aim to guide the enhancement of strategies and policies to better support APS workers as they work with clients and families at the end of life.

